# The evidence of metabolic-improving effect of metformin in *Ay/a* mice with genetically-induced melanocortin obesity and the contribution of hypothalamic mechanisms to this effect

**DOI:** 10.1371/journal.pone.0213779

**Published:** 2019-03-14

**Authors:** Kira Derkach, Irina Zakharova, Inna Zorina, Andrey Bakhtyukov, Irina Romanova, Liubov Bayunova, Alexander Shpakov

**Affiliations:** Department of Molecular Endocrinology and Neurochemistry, Sechenov Institute of Evolutionary Physiology and Biochemistry, Russian Academy of Sciences, St. Petersburg, Russia; Universidad Pablo de Olavide, SPAIN

## Abstract

In diet-induced obesity, metformin (MF) has weight-lowering effect and improves glucose homeostasis and insulin sensitivity. However, there is no information on the efficiency of MF and the mechanisms of its action in melanocortin-type obesity. We studied the effect of the 10-day treatment with MF at the doses of 200, 400 and 600 mg/kg/day on the food intake and the metabolic and hormonal parameters in female C57Bl/6J (genotype *A*^*y*^*/a*) agouti-mice with melanocortin-type obesity, and the influence of MF on the hypothalamic signaling in obese animals at the most effective metabolic dose (600 mg/kg/day). MF treatment led to a decrease in food intake, the body and fat weights, the plasma levels of glucose, insulin and leptin, all increased in agouti-mice, to an improvement of the lipid profile and glucose sensitivity, and to a reduced fatty liver degeneration. In the hypothalamus of obese agouti-mice, the leptin and insulin content was reduced and the expression of the genes encoding leptin receptor (LepR), MC_3_- and MC_4_-melanocortin receptors and pro-opiomelanocortin (POMC), the precursor of anorexigenic melanocortin peptides, was increased. The activities of AMP-activated kinase (AMPK) and the transcriptional factor STAT3 were increased, while Akt-kinase activity did not change from control C57Bl/6J (*a/a*) mice. In the hypothalamus of MF-treated agouti-mice (10 days, 600 mg/kg/day), the leptin and insulin content was restored, Akt-kinase activity was increased, and the activities of AMPK and STAT3 were reduced and did not differ from control mice. In the hypothalamus of MF-treated agouti-mice, the *Pomc* gene expression was six times higher than in control, while the gene expression for orexigenic neuropeptide Y was decreased by 39%. Thus, we first showed that MF treatment leads to an improvement of metabolic parameters and a decrease of hyperleptinemia and hyperinsulinaemia in genetically-induced melanocortin obesity, and the specific changes in the hypothalamic signaling makes a significant contribution to this effect of MF.

## Introduction

Biguanide metformin (MF) is the first-line pharmacologic agent for management of the type 2 diabetes mellitus and metabolic syndrome **[1]**. Acting on the peripheral tissues of diabetic individuals, the MF improves their sensitivity to insulin and reduces the plasma glucose levels due to both an inhibition of gluconeogenesis and a decrease of glucose production by hepatocytes **[1]**. Nowadays, the obtained clinical evidences show that the MF can be an effective drug for the treatment of both diabetic and non-diabetic patients with obesity **[2–4]**. The MF treatment of obese animals and patients has the weight- and fat-lowering effects and improves the glucose and insulin sensitivity **[5–8]**. The effectiveness of MF to treat the different types of obesity varies greatly and depends on the etiology of obesity, its severity and duration, as well as on the comorbid metabolic disorders, such as diabetes mellitus and metabolic syndrome. There is no evidence of a therapeutic effect of MF on the melanocortin-type obesity that is induced by chronic inhibition of the type 4 melanocortin receptor (MC_4_R). The weakening of hypothalamic MC_4_R-signaling can be caused by the reduced level of pro-opiomelanocortin (POMC), a precursor of the melanocortin peptides with MC_4_R-agonistic activity, by the increased levels of agouti-related peptide (AgRP) and agouti-signaling protein-1 (ASIP1), the endogenous MC_4_R antagonists, and by the impaired activity of MC_4_R due to inactivation mutations in the *Mc4r* gene **[9–17]**. Currently, the effective drugs to prevent and treat the melanocortin-type obesity are not developed **[18–20]**, which makes it necessary to develop the new pharmacological approaches for its correction, including the use of MF.

In the peripheral tissues, the main mechanism of MF action includes the inhibition of mitochondrial respiratory chain and the stimulation of AMP-activated protein kinase (AMPK), a crucial cellular energy sensor, which provides the regulation of metabolism, mitochondrial dynamics and endoplasmic reticulum stress **[21, 22]**. In the recent years, the greatest attention has been focused on the central mechanisms of MF action, which are based on its effect on the different brain structures, including the hypothalamus. The ability of MF to influence the brain functions and the neuronal plasticity determines the effectiveness of this drug in treating the neurological and neurodegenerative diseases **[23, 24]**. The MF is easily transferred to the hypothalamus and the other brain regions due to its efficient transport across the blood-brain barrier (BBB). As a result, the concentration of MF in the brain and the cerebrospinal fluid rapidly increases with different routes of its administration, as shown in the animal experiments **[25–28]** and in the clinical studies **[29]**. At the same time, in obesity the central mechanisms of MF action remain little investigated, and in the melanocortin-type obesity the mechanisms are not studied at all.

Nowadays, the hypothalamic signaling systems and, in the first place, the leptin signaling system are considered as the main targets of MF treatment, since their activity largely depends on the energy status of hypothalamic neurons. In the hypothalamus, leptin through leptin receptor (LepR) activates the 3-phosphoinositide and the STAT3 (signal transducer and activator of transcription 3) signaling pathways **[30]**. The 3-phosphoinositide pathway includes Janus kinase-2 (JAK2), insulin receptor substrate-2 (IRS2), phosphatidylinositol-3-kinase (PI3K) and serine/threonine-specific Akt kinase, the latter is responsible for control of neurogenesis and neuronal survival and growth **[31–33]**. The STAT3 pathway includes JAK2-mediated phosphorylation of LepR and subsequent phosphorylation and dimerization of STAT3, resulting in the activation of STAT3-dependent gene expression **[30, 34]**. In the hypothalamus, the 3-phosphoinositide and STAT3 pathways are involved in the functional interaction of the leptin system with the insulin, melanocortin and other signaling systems, which contributes to leptin-mediated regulation of food intake, energy expenditure and endocrine functions **[31, 33, 35–37]**. This is supported by the fact that PI3K and Akt-kinase are also the key enzymes in the brain insulin signaling **[38, 39]**. The PI3K, being a target for both leptin and insulin, integrates their regulatory effects on energy balance, glucose homeostasis, neurogenesis, neuronal survival and endocrine functions **[40]**. In diet-induced obesity the hypothalamic leptin and insulin signaling is largely changed, and these changes are the result of hormonal dysregulations and metabolic dysfunctions **[38, 41]**. We hypothesized that in melanocortin-type obesity the hypothalamic signaling systems also undergoes the significant changes, and anti-obesity effects of MF may include restoration and modulation of their activity.

The purpose was to study the effects of MF treatment of female yellow C57Bl/6J (genotype *A*^*y*^*/a*) agouti-mice (*Agouti yellow*) with the melanocortin-type obesity on their metabolic and hormonal parameters, as well as on the hypothalamic signaling. We investigated the influence of the 10-day treatment of obese agouti-mice with MF at three daily doses of 200, 400 and 600 mg/kg on the body weight, fat mass, food intake, glucose sensitivity, and the plasma levels of glucose, insulin, leptin and lipids. In the case of the most effective metabolic-improving dose (600 mg/kg/day), in the hypothalamus the activity of AMPK, STAT3 and Akt-kinase, as well as the gene expression for LepR, MC_3_R, MC_4_R and the anorexigenic and orexigenic factors which are involved in the regulation of food intake and energy expenditure were studied. We also investigated the effect of long-term administration of MF at a dose of 600 mg/kg/day on the functional state of the liver, evaluating its histology and the expression of the inflammatory and apoptotic factors, and also measured the plasma levels of lactate, which can be increased when using the high doses of MF **[42–44]**.

The choice of yellow C57Bl/6J (*A*^*y*^*/a*) mice with the mutation at the *Agouti* locus (*A*^*y*^) was due to the fact that they are the most suitable model of the melanocortin-type obesity. The agouti-mice (A^y^/a) have the increased expression of ASIP1, which antagonizes MC_4_R and provokes weight gain, fat deposition, glucose intolerance, the leptin and insulin resistance, and the impaired transport of leptin into the brain structures **[45–50]**.

In our study, it was first shown that the 10-day treatment of agouti-mice with melanocortin-type obesity with MF at the doses ranging from 200 to 600 mg/kg/day led to the decrease in the body and fat weight, food intake, dyslipidemia, hyperinsulinaemia and hyperleptinemia and to the restoration of the intrahypothalamic levels of leptin and insulin, and these effects were dose-dependent. In the hypothalamus of obese agouti-mice we detected the increase of the AMPK and STAT3 activities and the increased expression of the genes encoding LepR and the components of melanocortin signaling system, which can be considered as a compensatory response to obesity-associated metabolic abnormalities. At the most effective metabolic dose (600 mg/kg/day), the MF normalized the AMPK and STAT3 activities and increased the activity of Akt-kinase, the main effector enzyme of the 3-phosphoinositide pathway. Moreover, the MF treatment led to a significant increase in hypothalamic expression of the gene encoding POMC. It should be noted that MF at a dose of 600 mg/kg improved the liver functions, reducing the hepatic steatosis and normalizing the expression of pro-inflammatory and apoptotic factors in the liver tissue. These results indicate that MF should be considered as the effective drug to treat the melanocortin-type obesity, and give grounds to believe that specific changes in the leptin, melanocortin and insulin systems in the hypothalamus have an important role in the realization of the metabolic-improving effect of MF.

## Materials and methods

### Animals

In the experiments six-month female black C57Bl/6J (*a/a*) mice (control) and six-month female yellow C57Bl/6J (*A*^*y*^*/a*) mice (*Agouti yellow*) were used. The animals were housed in the plastic cages, ten animals in each, with a normal light–dark cycle (12 h/12 h, light on at 9.00 a.m.) and temperature (24±3°C) and had free access to laboratory chow pellets and water. The mice were obtained from the Institute of Cytology and Genetics of the Siberian Branch of the Russian Academy of Sciences. The experimental procedures were approved by the Institutional Animal Care and Use Committee at the Sechenov Institute of Evolutionary Physiology and Biochemistry, Russian Academy of Sciences, St. Petersburg, Russia, and according to “Guide for the Care and Use of Laboratory Animals” and to the European Communities Council Directive of 1986 (86/609/EEC). All efforts to minimize animal suffering and to reduce the number of animals were made.

Five groups of animals were investigated: black C57Bl/6J (*a/a*) mice (*n* = 15, Group a/a) that considered as control, obese yellow C57Bl/6J (*A*^*y*^*/a*) agouti-mice (*n* = 15, Group Ay/a) without MF treatment, and yellow C57Bl/6J (*A*^*y*^*/a*) agouti-mice treated with MF at the daily doses of 200 (*n* = 10, Group Ay/a-M200), 400 (*n* = 10, Group Ay/a-M400) and 600 (*n* = 15, Group Ay/a-M600) mg/kg for 10 days. The MF was dissolved in water and given to agouti-mice orally twice daily (at 10.00 and 18.00). In the Groups a/a and Ay/a, the animals received saline instead of MF solution. During the experiment, all the studied groups of mice received a standard chow diet that contained 19% protein, 4% fat, and 66% carbohydrates (“Assortment Agro”, Moscow Region, Turacovo, Russia), which provided 3.7 kcal/g. The blood samples to measure the levels of insulin, leptin, lipids and lactate were obtained from the heart under anesthesia (chloral hydrate, 400 mg/kg, intraperitoneally). Then, the anesthetized animals were decapitated, and the hypothalamus and liver were removed. The hypothalamus samples were used for qRT-PCR and Western blotting and for the determination of the intrahypothalamic leptin and insulin content, while the liver samples were used for qRT-PCR and histochemical analysis.

### The measurements of the plasma glucose, insulin, leptin, triacylglycerols, cholesterol and lactate levels and the intrahypothalamic leptin and insulin content

The glucose concentration in the whole blood from the tail vein was measured using a glucometer (“Life Scan Johnson & Johnson”, Denmark) and the test strips “One Touch Ultra” (USA). The concentration of insulin in the serum of mice and in the hypothalamus samples was measured using the Mouse Insulin ELISA kits (“Mercodia AB”, Sweden). The leptin levels in the serum and in the hypothalamus samples were measured using the ELISA kit for Leptin (“Cloud-Clone Corp.”, Houston, USA). To determine the intrahypothalamic leptin and insulin content, the samples of the hypothalamus tissue were homogenized in the ratio 1:10 in the lysis buffer containing 20 mM Tris-HCl (pH 7.5), 150 mM NaCl, 2 mM EDTA, 2 mM EGTA, 0.25 M sucrose, 0.5% Triton X-100, 0.5% sodium deoxycholate, 15 mM NaF, 10 mM sodium glycerophosphate, 10 mM sodium pyrophosphate, 1 mM Na_3_VO_4_, 1 mM phenylmethylsulfonyl fluoride (PMSF), 0.02% NaN_3_, and the protease inhibitor cocktail (“Sigma-Aldrich”, USA). The obtained homogenate was centrifuged (10 000 *g*, 5 min), and the concentration of leptin and insulin in the supernatant fraction was measured according to the manufacturer's instructions. The concentrations of total triglycerides and total cholesterol in the serum and the lactic acid levels in the blood plasma were measured using the enzyme colorimetric kits obtained from “Olvex Diagnosticum” (Russia).

### The glucose tolerance test

For glucose tolerance test (GTT), *D*-glucose (2 g/kg of the body weight) was intraperitoneally injected into the mice fasted during 6 h, and the plasma glucose levels were measured before (0 min) and 15, 30, 60, and 120 min after the glucose load. The area under the glucose concentration curve (AUC_0-120_) during the time interval from 0 to 120 min was calculated, as described earlier **[51]**. The GTT was performed two days before the end of experiment.

### Western blotting

The dissected hypothalamus tissues were homogenized in the ratio 1:20 in the lysis buffer containing 20 mM Tris-HCl (pH 7.5), 150 mM NaCl, 2 mM EDTA, 2 mM EGTA, 0.25 M sucrose, 0.5% Triton X-100, 0.5% sodium deoxycholate, 15 mM NaF, 10 mM sodium glycerophosphate, 10 mM sodium pyrophosphate, 1 mM Na_3_VO_4_, 1 mM phenylmethylsulfonyl fluoride (PMSF), 0.02% NaN_3_, and the protease inhibitor cocktail (“Sigma-Aldrich”, USA). The cell fragments and the undamaged cells were separated by centrifugation at 500 *g* for 10 min (4°C). The protein concentration was measured by the Lowry method with BSA as a standard. Thirty micrograms of protein per sample were run on 8% (the proteins with MW > 80 kDa) or 12% (the proteins with MW < 80 kDa) SDS-polyacrylamide gel, followed by transfer to a nitrocellulose membrane (0.45 μm) (“GE Healthcare, Amersham Biosciences AB”, United Kingdom) by electroblotting (300 mA, 1 h) in the mini trans-blot module (“Bio-Rad Laboratories Inc.”, USA). The non-specific binding was blocked in the TBST buffer containing 50 mM Tris-HCl (pH 7.5), 150 mM NaCl and 0.1% Tween 20 with the addition of 5% fat-free milk for 1 h at the room temperature. The membranes were incubated at 4°C overnight with the primary antibodies raised against phospho-Akt(Ser^473^) (1:1000) (#4085, “Cell Signaling Technology”, USA), phospho-Akt(Thr^308^) (1:1000) (#9275, “Cell Signaling Technology”, USA), phospho-AMPK-α(Thr^172^) (1:1000) (#2535, “Cell Signaling Technology”, USA), phospho-AMPK-α1(Ser^485^)/phospho-AMPK-α2(Ser^491^) (1:1000) (#4185, “Cell Signaling Technology”, USA), phospho-STAT3(Tyr^705^) (1:1000) (#9131, “Cell Signaling Technology”, USA). The immunostaining was made using the horseradish peroxidase-conjugated anti-mouse (#7076, “Cell Signaling Technology”, USA) or anti-rabbit (#7074, “Cell Signaling Technology”, USA) immunoglobulins at 1:1000–1:3000 dilution for 1 h at the room temperature, and the Novex ECL Chemiluminescent Substrate Reagent Kit (“Invitrogen, Life Technologies”, USA) and the premium X-ray film (“Phenix Research Product”, USA). To normalize the data, the membranes were treated with the antibodies raised against Akt-kinase (1:2000) (#9272, “Cell Signaling Technology”, USA), AMPK-α2 subunit (1:2000) (#NB100-238, “Novus Biologicals”, USA), STAT3 (1:1000) (#9139, “Cell Signaling Technology”, USA) and glyceraldehyde 3-phosphate dehydrogenase (GAPDH) (1:5000) (#NB600-502, “Novus Biologicals”, USA). The list of antibodies is present in the **S1 Table**. The relative amount of each protein was determined by adjusting for total protein (Akt-kinase, AMPK-α2, and STAT3) transferred to the blot or to GAPDH. The optical densities of the positive bands of the scanned films were quantified using the NIH Image Analysis software (“National Institutes of Health”, USA). A more detailed description of the Western blotting procedure is available at doi: http://dx.doi.org/10.17504/protocols.io.xqpfmvn

### The RNA extraction and qRT-PCR analysis

Total RNA was isolated from the sections of the hypothalamus and liver using the ExtractRNA Reagent (TRIzol analogue) (“Evrogen”, Moscow, Russia) according to the manufacturer‘s protocol. Prior to use, the RNA samples were examined by agarose gel electrophoresis to demonstrate clear bands corresponding to the ribosomal 5S/5.8S, 18S and 28S RNA and no degradation. The samples containing 1 μg of RNA were reverse-transcribed to cDNA using the MMLV RT kit (“Evrogen”, Moscow, Russia) and the random oligodeoxynucleotide primers. The PCR amplification was performed using the mixture (final volume of 25 μl) containing 10 ng of RT product, 0.4 μM each of the forward and reverse primers, and qPCRmix-HS SYBR+LowROX kit (“Evrogen”, Moscow, Russia). The amplified signals were detected continuously with the Applied Biosystems 7500 Real-Time PCR System (“Life Technologies, Thermo Fisher Scientific Inc.”, Waltham, Massachusetts, USA). The following qRT-PCR amplification protocol was used: (i) an initial denaturation at 95°C for 5 min; (ii) a 3-segment amplification and quantification program consisting of 38 cycles of 95°C for 30 s, 55–58°C for 10 s, and 72°C for 30 s; and (iii) the ABI Melt Curve program to verify the presence of a single peak and the absence of primer-dimer formation in each template-containing reaction. The annealing temperatures were optimized using the Primer-Blast program (http://www.ncbi.nlm.nih.gov/tools/primer-blast/). In the preliminary studies, the SYBR Green-labeled PCR products were evaluated by agarose gel electrophoresis, and the authenticity of each amplicon was verified by nucleic acid sequencing. The list of primers is present in the **S2 Table**. The data was calculated using the delta-delta C_t_ method and expressed as fold expression relative to the expression in control animals **[52]**. In the hypothalamus, the expression of the *Lepr*, *Mc3r* and *Mc4r* genes encoding LepR and MC_3_R and MC_4_R, and the expression of the *Pomc*, *Agrp* and *Npy* genes encoding the POMC, AgRP and neuropeptide Y (NPY) were estimated. In the liver, the expression of the *Bax*, *Bcl-2*, *IL1beta* and *TNFalpha* genes encoding pro-apoptotic factor Bax, anti-apoptotic factor Bcl-2 and the pro-inflammatory cytokines interleukin 1β and tumor necrosis factor-α (TNFα) were measured. The expression of the genes encoding 18S rRNA and hypoxanthine-guanine phosphoribosyl transferase (Hprt) was used as an endogenous control. A more detailed description of the qPCR procedure is available at doi: dx.doi.org/10.17504/protocols.io.xrnfm5e

### Histochemical analysis of the liver

After decapitation of anesthetized mice, the liver was removed and fixed using a buffered 10% formalin solution (pH 7.4) for 24 hours, and then the liver tissue was washed with 0.1 M Na^+^-phosphate buffer, pH 7.4. To prepare the liver sections the cryoprotection procedure was used. The liver tissue was immersed in a buffered solution containing 30% sucrose (+4°C, 48 h), and then frozen using dry ice and Tissue-Tek medium (“Sakura Finetek Europe”, Netherlands). Freezing liver was cut on a cryostat (“Leika Microsystems”, Germany), and the sections (7 μm thickness) were mounted on the Super Frost/Plus glasses (“Menzel”, Germany). The liver sections from animals of each of the studied groups were mounted on the same glass. According to standard histological procedures, the sections were stained with hematoxylin and eosin (“Labiko”, Russia), and after treatment with alcohol and xylene were placed into the BioMount medium (“Bio Optica”, Italy). In the case of the Sudan staining, the liver sections were washed sequentially with distilled water and 50% ethanol and then immersed in a solution of Sudan III (“Labiko”, Russia) for 15 min. After washing with distilled water and 50% ethanol, the Sudan-stained liver sections were stained with hematoxylin, washed with distilled water, placed under a cover glass using glycerol and used for histochemical analysis **[53].**

### Statistical analysis

All data were analyzed using the software IBM SPSS Statistics 22 (“IBM”, USA). An assumption of normality was assessed using the Kolmogorov-Smirnov test. The metabolic and hormonal data are presented as the weighted mean ± weighted standard deviation (*M ± SD*). The Western-blotting data are presented as the mean value *±* standard error of the mean (*M ± SEM*). The qRT-PCR data are presented as RQ (mean) *±* standard error of the RQ (mean) (*M ± SEM)*. The difference between the groups of mice was assessed statistically using the one-way analysis of variance (ANOVA, *t*-test) with the LSD (Least Significant Difference test) post hoc test and considered as significant at the *P <* 0.05.

## Results

### The effect of metformin treatment on the body and fat weight, food intake and lipid metabolism in obese agouti-mice

In the Group Ay/a, the body weight and the abdominal fat mass were increased significantly, despite the fact that the food intake did not differ significantly from the control animals (**Table 1 and S1 Dataset**), which corresponds to the data obtained earlier **[50]**. The treatment of obese agouti-mice with MF at all investigated doses led to a significant decrease in the body and fat weight. The MF treatment also reduced food intake, and in the Group Ay/a-M600 the food consumption was reduced by 18 and 14% as compared to the control and obese mice, respectively (**Table 1**). The plasma levels of total triglycerides and total cholesterol in the obese mice were increased significantly, and MF treatment at the dose of 600 mg/kg/day resulted in their normalization (**Table 1**). In agouti-mice treated by MF, including a daily dose 600 mg/kg/day, no mortality, indigestion, diarrhea and visible signs of pathological processes were observed. This data shows that MF is effective in reducing the body and fat weight and the food intake and is able to improve the lipid metabolism in genetically-induced melanocortin obesity.

**Table 1:** The body and fat weight, food intake and lipids in the obese agouti-mice and the effect of metformin treatment.

	a/a	Ay/a	Ay/a-M200	Ay/a-M400	Ay/a-M600
**Body weight (start), g** ^a^	23.1±1.5	37.4±1.8*	37.0±2.0*	37.1±2.1*	36.9±2.0*
**Body weight (final), g** ^b^	23.2 *±* 1.3	38.8 *±* 1.8* ^c^	32.5 *±* 1.4*# ^c^	32.3 *±* 2.5*# ^c^	32.0 *±* 1.8*# ^c^
**Abdominal fat mass, g**	0.41 *±* 0.05	3.99 *±* 0.25*	3.33 *±* 0.20*#	2.93 *±* 0.34*#	2.90 *±* 0.21*#
**Fat content, %**	1.78 *±* 0.17	10.31 *±* 0.76*	10.24 *±* 0.43*	9.07 *±* 0.69*#	9.06 *±* 0.49*#
**Food intake, g/day/mouse**^**&**^	2.89 *±* 0.22	2.75 *±* 0.17	2.46 *±* 0.22*#	2.43 *±* 0.19*#	2.36 *±* 0.19*#
**Triglycerides, mg/dL**	2.08 *±* 0.39	2.98 *±* 0.30*	2.59 *±* 0.33*#	2.26 *±* 0.31#	2.32 *±* 0.37#
**Total cholesterol, mg/dL**	4.02 *±* 0.64	4.74 *±* 0.57*	4.34 *±* 0.40	4.18 *±* 0.72#	4.06 *±* 0.39#

The data are presented as the *M ± SD*.

*—the difference between the Group a/a and all the groups of agouti-mice is significant at *P* < 0.05,

#—the difference between the Group Ay/a and the MF-treated agouti-mice (Ay/a-M200, Ay/a-M400 and Ay/a-M600) is significant at *P* < 0.05.

^&^—the averaged values for the food intake during 10 days. In each group: *n* = 10.

^a^ and ^b^–the body weight of mice at the beginning and at the end of the experiment, respectively.

^c^–the difference between the body weight at the beginning and at the end of the experiment in MF-treated groups is significant at *P* < 0.05.

### The effect of metformin treatment on the plasma glucose, insulin and leptin levels and the intrahypothalamic content of leptin and insulin in obese agouti-mice

In obese agouti-mice the plasma levels of fasting glucose and insulin were increased, and the treatment with MF at the dose of 600 mg/kg/day led to their decrease, while the doses of 200 and 400 mg/kg/day were less effective (**Table 2 and S1 Dataset**). The insulin resistance index calculated as the [fasting glucose, mmol/L]x[fasting insulin, ng/mL] in the obese mice was 6.7 times higher than in the control mice, and was reduced significantly in the agouti-mice treated with MF. In the Group Ay/a, the plasma leptin level was 8.7 times higher as compared to those in the control animals, and MF treatment reduced it (**Table 2**). Thus, the MF treatment led to the weakening of the peripheral hyperinsulinaemia and hyperleptinemia in obese agouti-mice, which indicates the partial restoration of the insulin and leptin sensitivity.

**Table 2:** The plasma levels of glucose, insulin and leptin and the intrahypothalamic leptin and insulin content in obese agouti-mice and the effect of metformin treatment.

	a/a	Ay/a	Ay/a-M200	Ay/a-M400	Ay/a-M600
**Fasting glucose, mmol/L**	5.2 *±* 0.6	10.0 *±* 1.6*	9.7 *±* 1.5*	8.7 *±* 1.0*#	8.1 *±* 1.2*#
**Plasma insulin, ng/mL**	0.32 *±* 0.10	1.09 *±* 0.40*	0.85 *±* 0.29*	0.88 *±* 0.32*	0.63 *±* 0.23*#
**Insulin resistance index**	1.67 *±* 0.58	11.13 *±* 4.79*	8.24 *±* 3.37*#	7.52 *±* 2.62*#	5.00 *±* 1.70*#
**Intrahypothalamic insulin, ng/g wet weight**	1.60 *±* 0.13	1.27 *±* 0.12*	ND	ND	1.49 *±* 0.10#
**The ratio of the intrahypothalamic/plasma insulin**	5.37 *±* 1.36	1.31 *±* 0.44*	ND	ND	2.68 *±* 1.03*#
**Plasma leptin, ng/mL**	1.58 *±* 0.32	13.74 *±* 3.98*	9.83 *±* 2.47*#	9.48 *±* 2.52*#	7.20 *±* 1.49*#
**Intrahypothalamic leptin, ng/g wet weight**	12.89 *±* 1.98	9.80 *±* 1.08*	11.08 *±* 0.98*	11.73 *±* 1.20#	11.80 *±* 1.93#
**The ratio of the intrahypothalamic/plasma leptin**	8.43 *±* 1.87	0.78 *±* 0.28*	1.20 *±* 0.33*	1.35 *±* 0.50*	1.71 *±* 0.49*#

The data are presented as the *M ± SD*.

*—the difference between the Group a/a and all the groups of agouti-mice is significant at the *P* < 0.05,

#—the difference between the untreated and MF-treated agouti-mice is significant at the *P* < 0.05. In each group: *n* = 10. ND, No Detection.

In contrast to the peripheral hyperleptinemia and hyperinsulinaemia, in obese mice the intrahypothalamic content of leptin and insulin was reduced by 24 and 21%, respectively, as compared to the control mice (**Table 2**). The decreased levels of leptin and insulin in the hypothalamus of obese agouti-mice, we believe, are the result of the impaired receptor-mediated transport of leptin and insulin across the BBB in the conditions of the leptin and insulin resistance. The MF treatment reduces the peripheral hyperleptinemia and hyperinsulinaemia and normalizes the transport of leptin and insulin through the BBB. The ratio intrahypothalamic/plasma leptin in the Group Ay/a was 11 times lower than in the control mice, and in the Groups Ay/a-M200, Ay/a-M400 and Ay/a-M600 this ratio was increased by 54, 73 and 119%, respectively. The ratio intrahypothalamic/plasma insulin in the obese agouti-mice was four times lower than in control C57Bl/6J (*a/a*) mice, and MF (600 mg/kg/day) led to increase of this ratio by 105% (**Table 2**).

### The glucose tolerance in obese agouti-mice and the effect of metformin treatment

**Fig 1:**
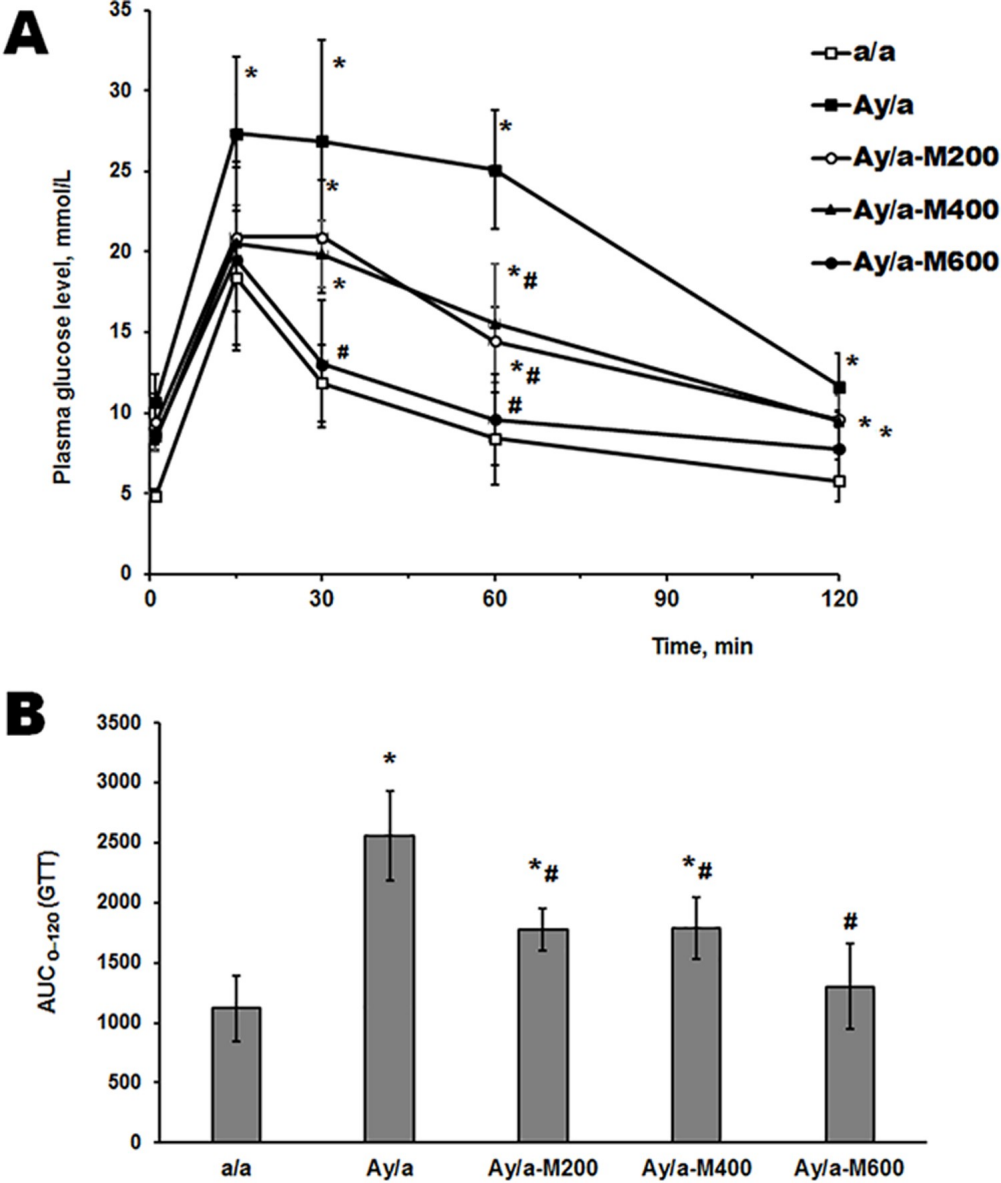
Glucose curves and AUC_0-120_ in GTT in obese agouti-mice and the effect of metformin treatment. (A) The glucose concentration curves in the glucose tolerance test. (B) The AUC_0-120_ values for the glucose concentration curves during the time interval from 0 to 120 min. The data are presented as the *M ± SD*. *—the difference between the control mice and all the studied groups of agouti-mice is significant at *P* < 0.05; #—the difference between the Group Ay/a and MF-treated agouti-mice is significant at *P* < 0.05. In each group: *n* = 5.

Using the GTT, it was shown that in obese agouti-mice the glucose tolerance was impaired, which was illustrated by the increased glucose concentration 30, 60 and 120 min after the glucose load and by the elevated AUC_0-120_ value calculated for area under the glucose concentration curve (**Fig 1A and S2 Dataset**), which is consistent with previously obtained data **[50]**. The MF treatment of obese animals led to the improvement of the glucose sensitivity, and the dose of 600 mg/kg/day was the most effective. The glucose levels 60 and 120 min after the glucose load and the AUC_0-120_ values in MF-treated obese mice were significantly decreased in comparison with the Group Ay/a and did not differ from control (**Fig 1B and S2 Dataset)**.

### The effect of high-dose metformin on the functional state of the liver and the plasma lactate levels in obese agouti-mice

The above data indicates that the most pronounced improving effect of MF on the metabolic and hormonal parameters in obese agouti-mice was obtained when the MF was used at the high dose of 600 mg/kg/day. At the same time, there is evidence that high-dose MF can induce the histological and biochemical changes in the liver, and can increase the plasma lactate levels, inducing lactic acidosis **[42–44]**. To study the influence of high-dose MF on liver functions in the melanocortin-type obesity, we studied the liver histology and the hepatic expression of the genes encoding the pro-inflammatory and apoptotic proteins and also measured the plasma lactate levels in the Groups Ay/a and Ay/a-M600.

**Fig 2:**
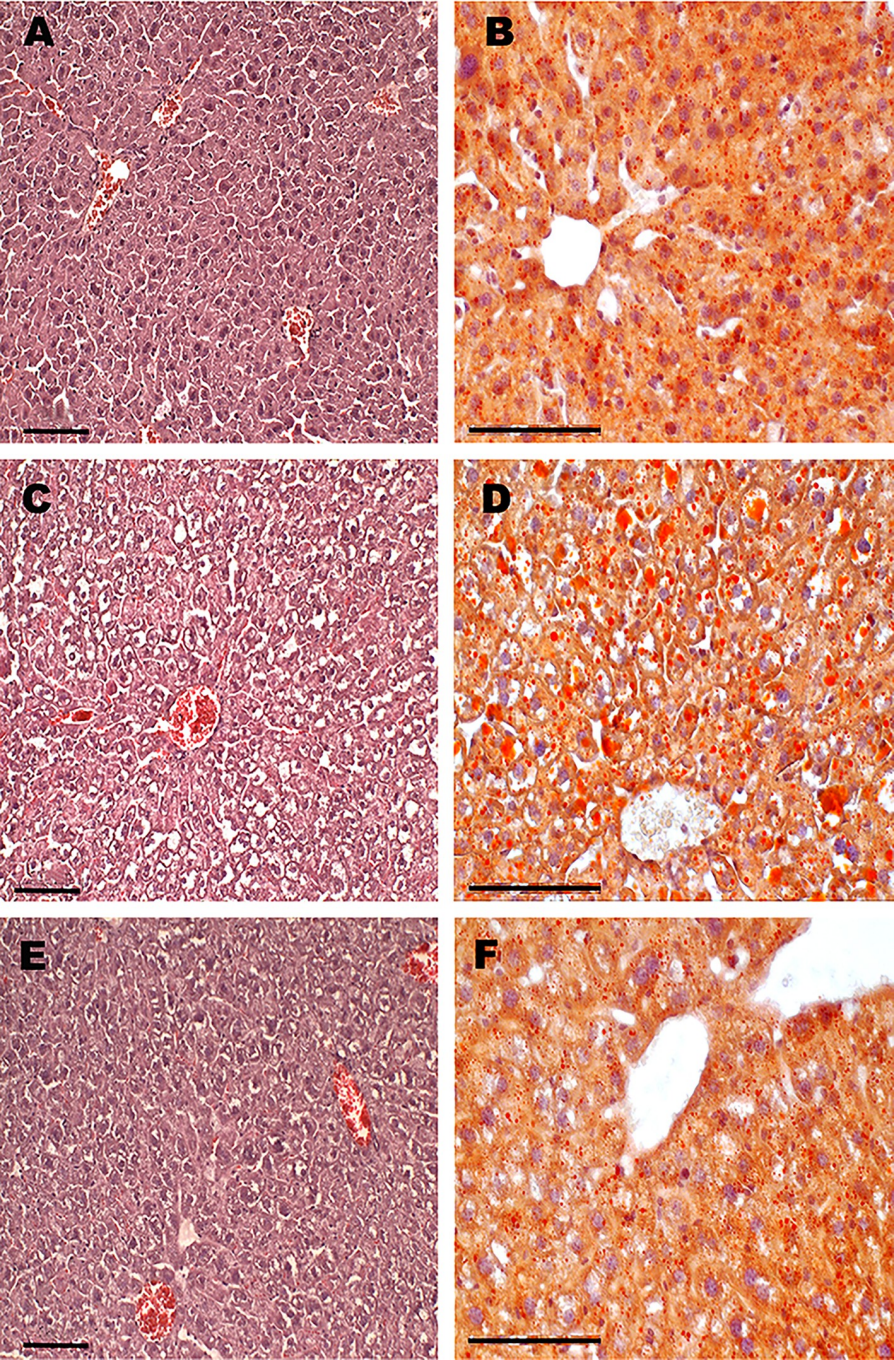
The histological evidence of steatosis in the liver of obese agouti-mice, and the improving effect of long-term metformin treatment (600 mg/kg/day). A, B–Group a/a (control); C, D–Group Ay/a; E, F–Group Ay/a-M600. The liver histology was evaluated using the staining of liver sections with hematoxylin and eosin (A, C, E), while the hepatic lipid deposition was evaluated by the Sudan III-staining (B, D, F). The orange inclusions and granules after the Sudan III staining are the lipid and lipoproteins accumulations located within hepatocytes. The liver sections from animals of each of the three studied groups were mounted on the same glass. Scale bars, 100 μm.

A histochemical analysis of the liver sections obtained from the obese agouti-mice and control animals showed that in agouti-mice there were signs of fatty liver dystrophy and vacuolar degeneration of hepatocytes that are typical for non-alcoholic fatty liver disease (**Fig 2**). The size of hepatocytes and the cytopasm/nucleus ratio in them were increased, and some hepatocytes had nuclei localized at the periphery rather than in the central part of the cell (**Fig 2C**). In the hepatocytes of agouti-mice, a total number of Sudan-positive vacuoles was 3–4 times higher than in the control mice (**Fig 2B and 2D**). In MF-treated agouti-mice, the liver degeneration and vacuolar degeneration of hepatocytes were significantly less pronounced as compared to the Group Ay/a (**Fig 2**). The number of Sudan-positive lipid inclusions in hepatocytes was only 1.5–2 times higher than in controls (**Fig 2B and 2F**), and the location of the nuclei in hepatocytes was predominantly central (**Fig 2**). Thus, the 10-day treatment with MF (600 mg/kg/day) significantly reduced the obesity-associated pathological changes in the liver of agouti-mice.

**Fig 3:**
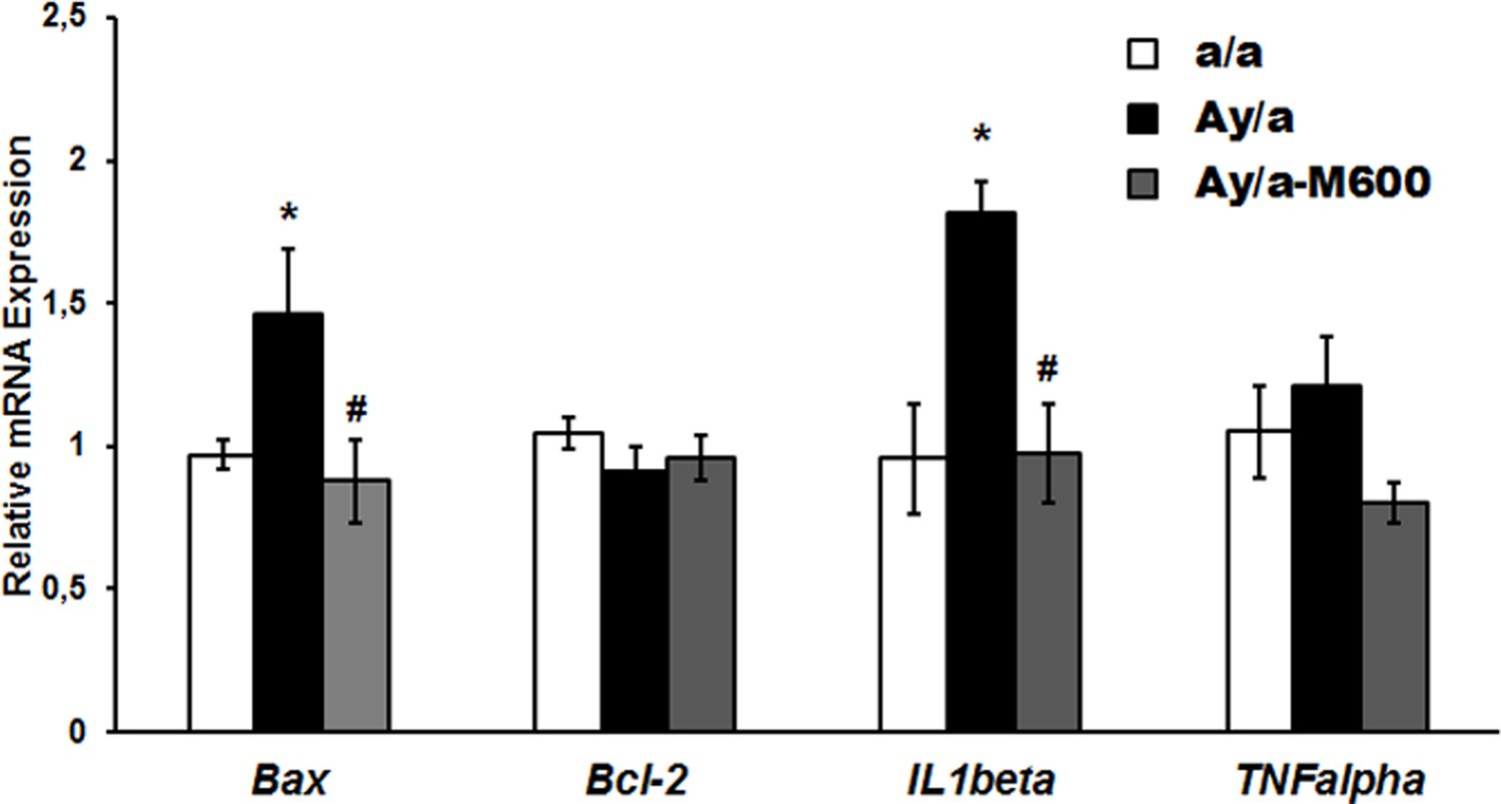
The gene expression of the proteins involved in apoptosis and inflammation in the liver of obese agouti-mice and the effect of metformin treatment. The levels of mRNA expression of the *Bax*, *Bcl-2*, *IL1beta* and *TNFalpha* genes encoding pro-apoptotic factor Bax, anti-apoptotic factor Bcl-2, the inflammatory cytokines interleukin 1β and TNFα are normalized by the expression of the reference *18s rRNA* gene. The relative mRNA expression is calculated with the respect to control group. The data are presented as the *M ± SEM*, *n* = 5. *—the difference between the Groups a/a and Ay/a-M600 is significant at the *P* < 0.05, #—the difference between the Groups Ay/a and Ay/a-M600 is significant at the *P* < 0.05.

The expression of the *Bax* gene in the liver of obese agouti-mice was increased by 51% as compared to the Group a/a, and it was decreased significantly in MF-treated agouti mice (**Fig 3 and S2 Dataset**). The Bax/Bcl-2 ratio was also increased by 72%, and the difference was significant as compared to the control animals (1.62±0.24 *vs*. 0.94±0.07, *P*<0.05). The MF treatment returned the Bax/Bcl-2 ratio to that in the control animals, and this ratio was differed significantly from that in agouti mice (0.92±0.15, *P*<0.05 as compared with the Group Ay/a), which indicates a normalization of the Bax-mediated apoptosis in the liver of agouti-mice treated with MF. In the Group Ay/a, the expression of the *IL1beta* gene encoding pro-inflammatory interleukin 1β was increased by 90% (*P*<0.05 as compared to control), while in the Group Ay/a-M600 the *IL1beta* gene expression was similar to that in the control animals (**Fig 3**). The expression of the *TNFalpha* gene in agouti-mice did not differ significantly from control. In the Group Ay/a-M600, the *TNFalpha* expression was decreased as compared with the Group Ay/a, but the difference was not significant (*P* = 0.062) (**Fig 3**). These data demonstrate a decrease in the gene expression of the main pro-inflammatory cytokines in the liver of agouti-mice treated with high-dose MF.

The plasma level of lactic acid in agouti-mice was higher than in the control mice, but the difference was not significant (3.53±0.82 *vs*. 2.64±0.65 mM, *P*>0.05), and long-term treatment of agouti-mice with MF (600 mg/kg/day) led to an increase in lactate level by 129% as compared to untreated animals (8.08±1.90 mM, 16 hours after the final administration of MF) (*P*<0.05 as compared to both the Groups a/a and Ay/a) (**S1 Dataset**). These data indicated that the dose 600 mg/kg/day led to a mild increase in the plasma lactate level and, therefore, did not cause the severe lactic acidosis. It should be noted that when MF was administered to agouti-mice for one day at the same dose, the plasma level of lactate was increased to 6.95±1.47 mM, which did not differ from those in agouti-mice after 10-day MF treatment (*P*>0.05). These results indicate that there is no effect of lactate accumulation in the blood of obese agouti-mice in the conditions of long-term administration of high-dose MF.

### The activity of AMPK, Akt-kinase and STAT3 in the hypothalamus of obese agouti-mice and the effect of metformin

**Fig 4:**
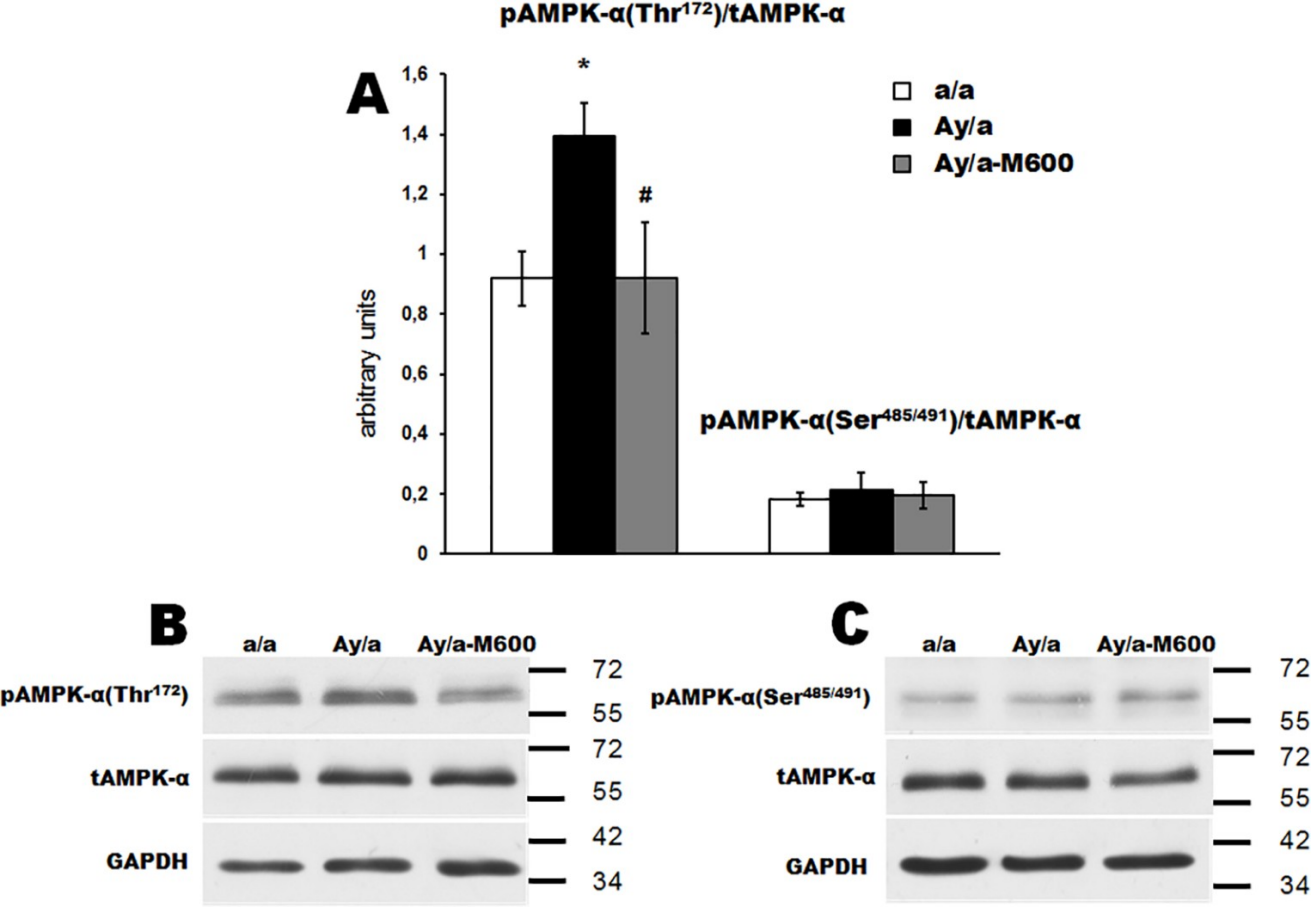
Phosphorylation of AMPK α-subunits in the hypothalamus of obese agouti-mice and the effect of metformin treatment. (A) The histograms for the ratio of the Thr^172^- and Ser^485/491^-phosphorylated α1/2-AMPK and the non-phoshorylated α2-AMPK. (B) and (C) The Western blotting for the Thr^172^- and Ser^485/491^-phosphorylated forms of α1/2-AMPK, respectively. The data are presented as the *M ± SEM*. *—the difference between the Groups a/a and Ay/a is significant at the *P* < 0.05, #—the difference between the Groups Ay/a and Ay/a-M600 is significant at the *P* < 0.05. In each group: *n* = 5.

The AMPK is the target of MF both at the periphery and in the CNS **[21–23]**, and the phosphorylation of its α1/2-subunits at the Thr^172^ leads to the activation of AMPK, while the phosphorylation of the α1/2-subunits at the Ser^485/491^ is the regulatory mechanism inducing the decrease in AMPK activity **[23, 54, 55]**. We showed that the Thr^172^-phosphorylation of α1/2-AMPK in the hypothalamus of obese mice was increased significantly, while the Ser^485/491^-phosphorylation did not change (**Fig 4 and S2 Dataset**). The treatment with MF (600 mg/kg/day) led to a decrease in the Thr^172^-phosphorylation, but did not affect the Ser^485/491^-phosphorylation, normalizing, thus, AMPK activity in the hypothalamus of MF-treated animals.

**Fig 5:**
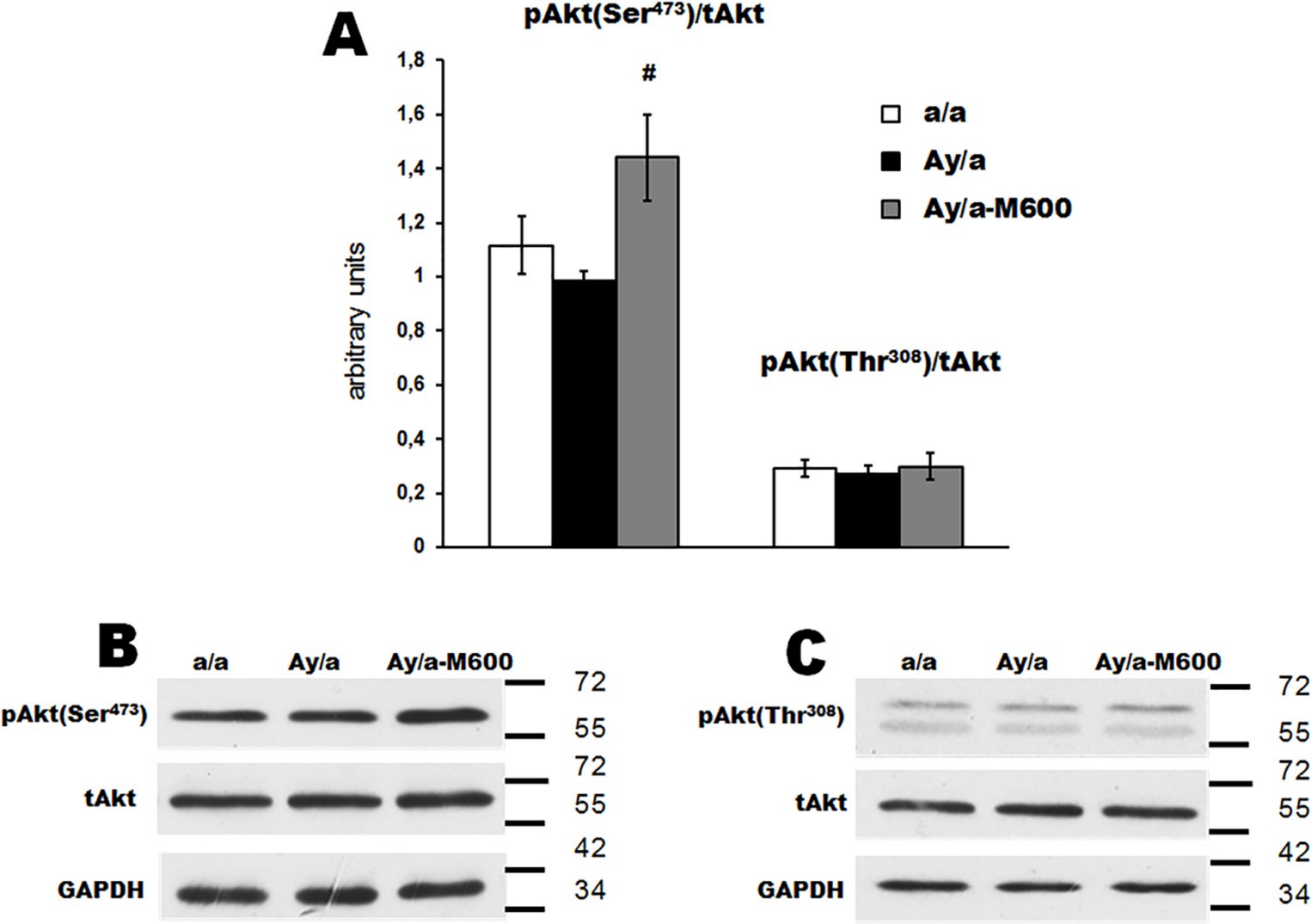
Phosphorylation of Akt-kinase in the hypothalamus of obese agouti-mice and the effect of metformin treatment. (A) The histograms for the ratio of the Ser^473^- and Thr^308^-phosphorylated and non-phosphorylated Akt-kinase. (B) and (C) The Western blotting for the Ser^473^- and Thr^308^-phosphorylated forms of Akt kinase, respectively. The data are presented as the *M ± SEM*. #—the difference between the Groups Ay/a and Ay/a-M600 is significant at the *P* < 0.05. In each group: *n* = 5.

**Fig 6:**
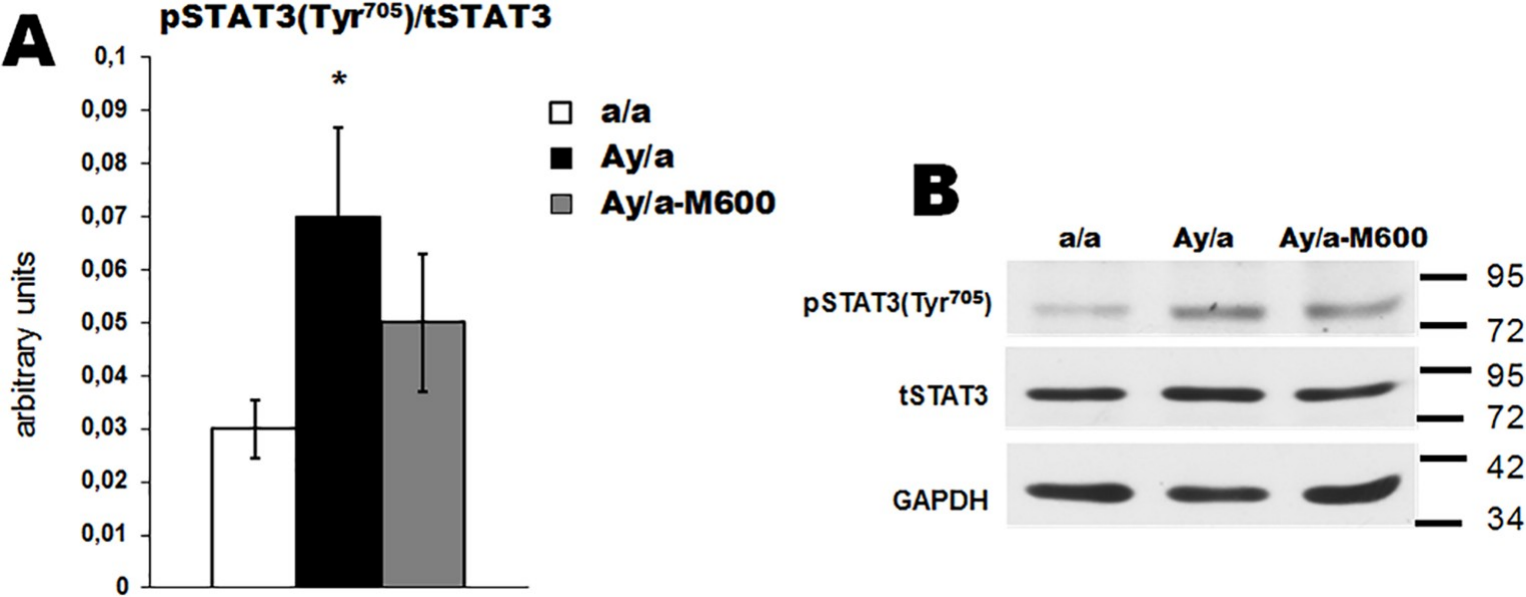
Phosphorylation of STAT3 in the hypothalamus of obese agouti-mice and the effect of metformin treatment. (A) The histograms for the ratio of the Tyr^705^-phosphorylated and non-phosphorylated STAT3. (B) The Western blotting for the Tyr^705^-phosphorylated STAT3. The data are presented as the *M ± SEM*. *—the difference between the Groups a/a and Ay/a is significant at the *P* < 0.05. In each group: *n* = 5.

In the hypothalamus, the Akt-kinase, the main component of the 3-phosphoinositide pathway, is activated by leptin (LepR/JAK2/IRS2/PI3K/Akt) and insulin (insulin receptor/IRS2/PI3K/Akt), while the transcriptional factor STAT3 is the downstream component of leptin-regulated LepR/JAK2/STAT3 pathway **[30, 31, 34, 39, 40, 56]**. The activation of the 3-phosphoinositide pathway by leptin and insulin leads to an increase in the Ser^473^- and Thr^308^-phosphorylation of Akt-kinase, and the activation of STAT3 by leptin induces an increase in the Tyr^705^-phosphorylation of STAT3. We showed that in the hypothalamus of agouti-mice, the Ser^473^- and Thr^308^-phosphorylation of Akt kinase did not differ from the control (**Fig 5 and S2 Dataset**), while the Tyr^705^-phosphorylation of STAT3 was increased by 116% (**Fig 6 and S2 Dataset**). The MF treatment led to a significant increase of the Ser^473^-phosphorylated Akt kinase, which indicates the activation of this enzyme (**Fig 5**). Alongside, the Tyr^705^-phosphorylated STAT3 in the Group Ay/a-M600 was decreased and did not differ significantly from control (**Fig 6**). Thus, in the hypothalamus of untreated agouti-mice the STAT3 pathway was hyperactivated, while MF treatment led to the normalization of STAT3 activity and to the strengthening of the 3-phosphoinositide signaling.

### The expression of the genes encoding the leptin and melanocortin receptors and the orexigenic and anorexigenic factors in the hypothalamus of agouti-mice, and the effect of metformin

**Fig 7:**
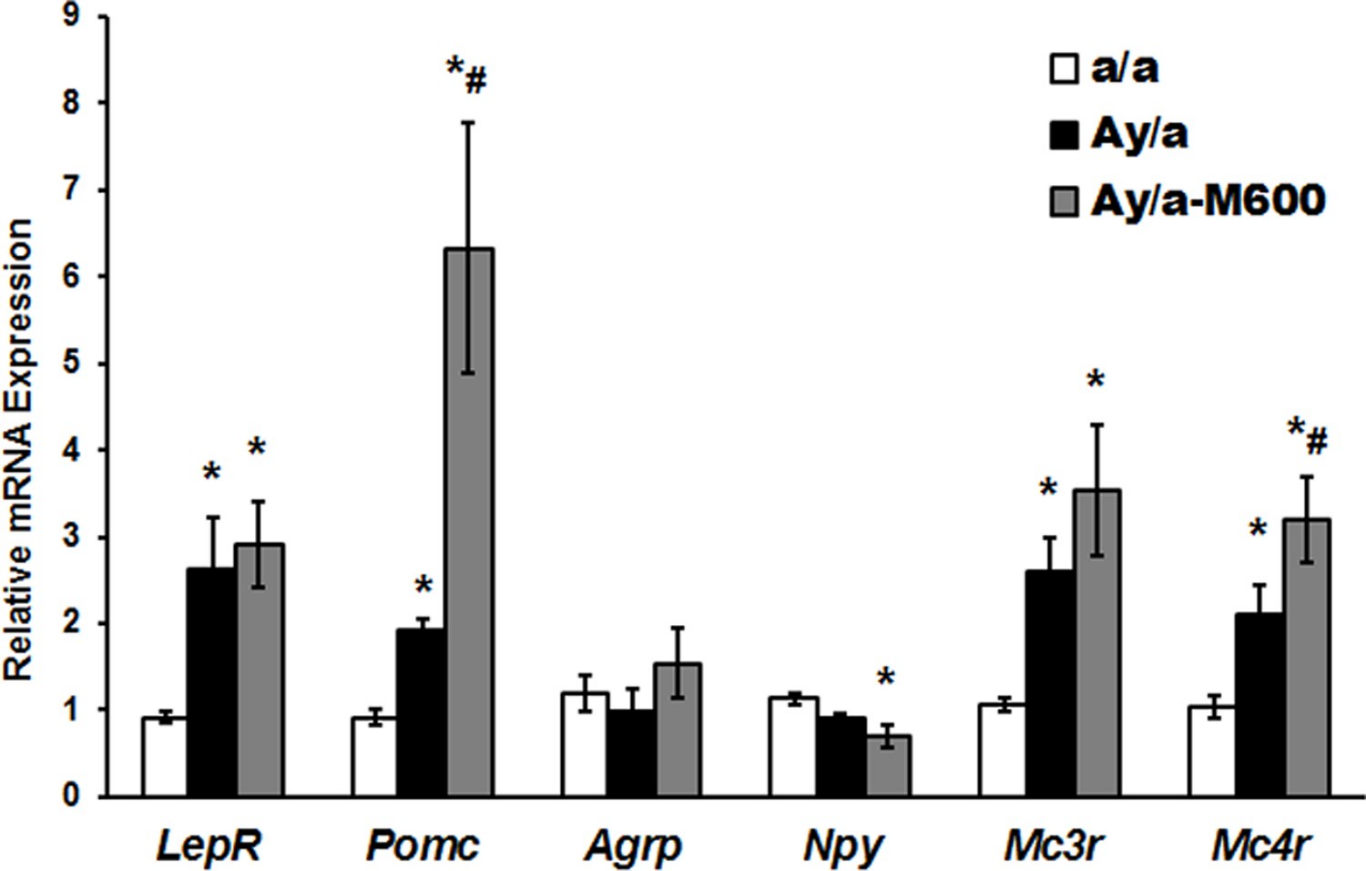
The expression of the genes in the hypothalamus of obese agouti-mice and the effect of metformin treatment. The levels of mRNA expression of the *Lepr*, *Pomc*, *Agrp*, *Npy*, *Mc3r* and *Mc4r* genes encoding LepR, POMC, AgRP, NPY, MC_3_R and MC_4_R are normalized by the expression of the reference *18s rRNA* and *Hprt* genes. The relative mRNA expression is calculated with the respect to control group. The data are presented as the *M ± SEM*, *n* = 5. *—the difference between the Groups a/a and Ay/a-M600 is significant at the *P* < 0.05, #—the difference between the Groups Ay/a and Ay/a-M600 is significant at the *P* < 0.05.

In the hypothalamus, the expression of the genes encoding the receptor components of the leptin (LepR) and melanocortin (MC_3_R and MC_4_R) signaling systems and the orexigenic (AgRP, NPY) and pro-anorexigenic (POMC) factors was estimated by qRT-PCR analysis. We showed a significant increase in the *Lepr*, *Pomc*, *Mc3r* and *Mc4r* genes expression in obese agouti-mice as compared to control (**Fig 7 and S2 Dataset**). The MF treatment led to an increase in this expression even more than in the Group Ay/a, although a significant difference between the Groups Ay/a and Ay/a-M600 was detected only for the *Pomc* expression. The *Pomc* expression in the Group Ay/a-M600 was six and three times higher than in the Groups a/a and Ay/a, respectively (**Fig 7**). The expression of the *Npy* gene had tendency to a decrease in the Group Ay/a, and it was significantly decreased in the Group Ay/a-M600. Meanwhile, the expression of the *Agrp* gene encoding the orexigenic factor AgRP did not change significantly in all investigated groups (**Fig 7**).

## Discussion

**Fig 8:**
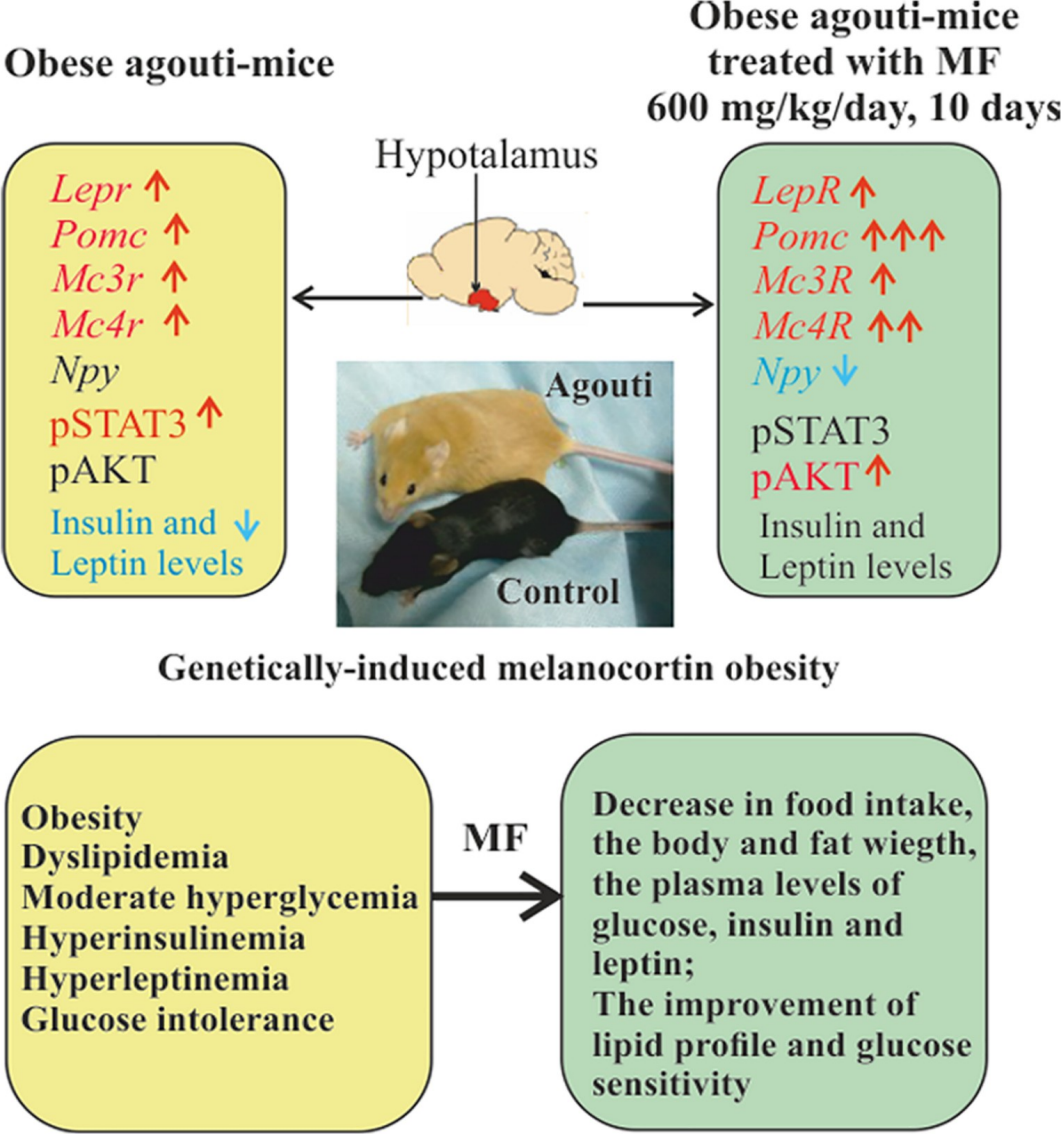
Metabolic and hormonal parameters and hypothalamic regulation in obese agouti-mice and the effect of metformin treatment.

The results obtained by us indicate that the agouti-mice with the “yellow” mutation at the mouse agouti locus (*A*^*y*^) associated with the overexpression of ASIP1, the endogenous antagonist of the MC_1_R and MC_4_R had the increased body weight and fat mass, the elevated levels of glucose, insulin and leptin, the impaired glucose tolerance and the altered lipid metabolism (**Fig 8**). These data indicate the decreased glucose, insulin and leptin sensitivity and the signs of dyslipidemia in MC_4_R signaling-deficient animals, and are in a good agreement with the previously obtained results on metabolic abnormalities and hyperleptinemia in obese agouti-mice **[49, 50, 57]**.

In the agouti-mice, the main cause of metabolic abnormalities described above is the chronic ASIP1-induced inhibition of hypothalamic MC_4_R-mediated signaling that regulates the food intake, energy expenditure, insulin sensitivity and the carbohydrate and lipid metabolism **[45–48, 50, 57]**. This conclusion is supported by the data of other researchers on the close relationship between dysfunctions in the hypothalamic MC_4_R-melanocortin system and the development of obesity, insulin resistance and other metabolic abnormalities **[10, 11, 14, 58]**. In clinical studies of patients with obesity and type 2 diabetes mellitus, it was demonstrated that some of them had the impaired hypothalamic MC_4_R-signaling and the inactivating mutations within the *Mc4r* gene **[12, 16, 59, 60]**. The treatment of mice with MC_4_R antagonists and the knockout of the *Mc4r* gene in the hypothalamus led to hyperphagia, obesity, insulin resistance and reduced energy expenditure, while the treatment of obese rodents with MC_4_R agonists and the restoration of the *Mc4r* gene expression in the paraventricular hypothalamus of the *Mc4r*^*-/-*^ mice resulted in the normalization of their body and fat weight, the decrease in appetite and the improvement of the glucose and insulin sensitivity **[9–11, 13–15]**. The obese *Mc4r*^*-/-*^ mice had the strongly pronounced hyperleptinemia **[9]** and did not respond to the inhibitory effects of leptin on feeding **[61]**.

Moreover, it is commonly believed that the *Mc4r*^*-/-*^ mice are considered as an appropriate model of non-alcoholic fatty liver disease with its typical features, such as the hepatic steatosis and the increased activity of the pro-inflammatory and apoptotic factors **[62, 63]**. These data are in a good agreement with our results on the liver dysfunctions in agouti-mice. We detected the fatty liver dystrophy, vacuolar degeneration of hepatocytes and the increased expression of factors responsible for inflammation and apoptosis in the liver of obese agouti-mice, which indicates the metabolic dysregulations in the hepatocytes and, along with the insulin and leptin resistance is considered as an important mechanism of triggering the non-alcoholic fatty liver disease **[64–66]**.

We showed that obese agouti-mice had the decreased intrahypothalamic levels of leptin and insulin, which, we believe, is mainly due to the disruption of their receptor-mediated transport across the BBB in the conditions the peripheral leptin and insulin resistance. It should be noted that a decrease in the intrahypothalamic leptin level in the agouti-mice was shown by us earlier **[50]**, while a decrease in the intrahypothalamic insulin level in the melanocortin obesity was shown by us for the first time. In another work, Pan and coauthors showed that in 8-month old agouti-mice with pronounced hyperleptinemia the permeation of the plasma leptin through the BBB was slower as compared to the control B6 mice, despite the increased level of ObRa, the main transport form of LepR, in astrocytes and cerebral microvessels **[67]**. The reduced insulin level in the cerebrospinal fluid was observed in rats with type 2 diabetes mellitus associated with obesity and hyperinsulinaemia **[68]**.

In the hypothalamus, leptin and insulin positively regulate the production of POMC, the precursor of anorexigenic melanocortin peptides, and suppress the production of orexigenic AgRP, the endogenous MC_4_R antagonist, controlling, thus, melanocortin-dependent food intake and energy expenditure **[39, 40, 56, 69–76]**. The inhibition of hypothalamic leptin system, as a result of the decrease in intracerebral leptin level in the fasting conditions and the knockout of the genes encoding leptin and LepR, leads to the reduced expression of the *Pomc* gene and the increased expression of the *Agrp* gene within the arcuate nuclei of hypothalamus **[69]**. The intracerebroventricular administration of leptin results in both the increased expression of the genes encoding POMC and MC_4_R and the decreased expression of the *Agrp* gene **[71]**. Leptin controls the production of POMC and AgRP by hypothalamic neurons through the 3-phosphoinositide- and STAT3-dependent pathways, while insulin realizes this effect mainly through the 3-phosphoinositide pathway **[39, 40, 56, 77–81].** It was shown that the leptin-induced activation of LepR located on the POMC/CART- and AgRP/NPY-neurons leads to phosphorylation and dimerization of STAT3, translocation of homodimeric STAT3-complex into the nucleus and its interaction with promoter regions of the *Pomc* and *Agrp* genes **[81]**. The inhibition of 3-phosphoinositide pathway by PI3K antagonists and the specific knockout of gene encoding the regulatory p85-PI3K subunit in POMC/CART neurons lead to the suppression of the stimulating effect of leptin on the *Pomc* expression **[72]**. It should be noted that the PI3K is the most important integrating component of the leptin and insulin signaling pathways responsible for regulating the activity of the POMC/CART- and AgRP/NPY-neurons. Moreover, in the hypothalamus insulin through PI3K induces the activation of the leptin signaling, and this is a molecular mechanism that provides a synergistic action of insulin and leptin on food intake and energy expenditure. Recently, it was shown that the long-term disruption of the genes encoding p110α and p110β, the catalytic subunits of PI3K, within the AgRP/NPY-neurons abrogates the leptin- and insulin-induced inhibition of AgRP/NPY-neurons and lead to the strengthening of orexigenic regulations, hyperphagia and obesity **[40]**.

In our experiments, the expression of the *Lepr* gene in the hypothalamus of obese agouti-mice was increased significantly, which is a compensatory mechanism to restore the leptin signaling in the conditions of the decreased intrahypothalamic level of leptin and insulin. In our view, the increased *Lepr* expression, at least in part, ensures the preservation (Akt-kinase) or enhancement (STAT3) of activity of the effector components of the leptin signaling, which illustrated by the increased Tyr^705^-phosphorylation of STAT3 and the unchanged Thr^308^- and Ser^473^-phosphorylation of Akt-kinase in the hypothalamus of obese animals. As a result, the expression of the *Pomc* gene was increased two-fold (**Figs 5–8**). This provides the increased production of the melanocortin peptides required for activation of MC_4_R in the conditions of chronic ASIP1 overexpression. On the other hand, it should be noted that a prolonged increase in the STAT3 activity is not sufficient to promote the POMC expression. Moreover, the hyperactivated STAT3 induces the suppression of the 3-phosphoinositide pathway and, as a result, provokes the leptin and insulin resistance in the hypothalamus, as was shown in mice expressing a constitutively active STAT3 in the POMC-neurons **[82]**.

The other compensatory mechanism includes the two-fold increase of the *Mc3r* and *Mc4r* expression in the hypothalamus of obese agouti-mice (**Figs 7 and 8**). The most interesting is an increase in the *Mc3r* expression, since hypothalamic MC_3_R functions as an autoreceptor involved in feed back regulation of the MC_4_R-signaling. There are grounds for believing that in the conditions of the ASIP1-antagonized MC_4_R-signaling, the functions of MC_4_R can be forwarded to the MC_3_R, as was demonstrated in the *Mc4r*^−/−^ mice and in obese rats with chronic autoimmune inhibition of MC_4_R **[38, 83]**. It was shown that the anorectic effect of melanotan-II, a mixed MC_3_R/MC_4_R agonist injected into the *Mc3r*^−/−^ or *Mc4r*^−/−^ mice was maintained, although it was significantly decreased, while in the case of a double knockout of these genes this effect was not detected **[83]**. Earlier we showed that immunization of rats with the BSA-conjugated peptide homologous to the extracellular N-terminal region of MC_4_R, inducing chronic inhibition of the MC_4_R-signaling led to the enhancement of intrahypothalamic MC_3_R signaling **[38]**. This indicates that the MC_3_R is able to interchange the MC_4_R, at least to a certain extent, which, we believe, takes place in obese agouti-mice.

Despite the increased body weight and fat mass and the signs of dyslipidemia, the food consumption in the obese agouti-mice did not differ significantly from the control animals. We supposed that this fact was due to the reduced energy expenditure in adult obese animals with the impaired hypothalamic MC_4_R-signaling. The other authors demonstrated that the *Mc4r*^*-/-*^-mice consumed less oxygen than control animals **[14, 84]**. Moreover, the decrease in the metabolic rate in the knockout mice was detected before they had an increased body weight and metabolic abnormalities. In the satiety state, the *Mc4r*^*-/—*^mice had an increased respiratory rate, which indicated the enhanced utilization of carbohydrates due to lower fat oxidation, and, eventually, led to the accumulation of the adipose tissue **[85–87]**. Thus, we believe that the main cause of obesity and fat deposit in agouti-mice is a decrease in the rate of energy metabolism, as result of the weakening of hypothalamic MC_4_R-signaling.

The 10-day treatment of obese agouti-mice with MF at the dose of 600 mg/kg/day led to the decrease in the body and fat weight, food intake and plasma levels of insulin and glucose and to the improvement of insulin sensitivity, which demonstrates the efficiency of MF to treat the melanocortin-type obesity (**Fig 8**). The use of the lower daily doses, 200 and 400 mg/kg, also had the weight- and fat-lowering effects, reduced the food intake and partially restored the metabolic parameters, but was less effective than the dose of 600 mg/kg. Earlier, the improving effect of MF on the body and fat weight and the metabolic parameters was demonstrated in animals with the other types of obesity and in obese patients **[2–8]**.

The increase of the insulin sensitivity and the restoration of lipid metabolism is the main mechanism of weight-lowering effect of MF. At the periphery, this mechanism is realized primarily due to the ability of MF to inhibit the mitochondrial respiratory chain and activate AMPK **[21, 22]**. In the recent years, the strong evidences were obtained that the important target for MF is the hypothalamus, and its effects on the hypothalamic signaling makes an important contribution to MF-induced improvement of the metabolic processes and the peripheral insulin and leptin sensitivity in obesity and other metabolic disorders **[23]**. In the hypothalamus, unlike the peripheral tissues, the MF does not activate and even suppresses AMPK activity, which leads to the activation of thermogenesis and decreases the body weight and fat deposition **[54, 55]**.

The effects of MF on appetite and food consumption can be realized through both the AMPK-dependent and AMPK-independent mechanisms. It must be emphasized that in the CNS, the MF does not increase, as at the periphery, but suppresses the AMPK activity **[23]**. It was shown that MF-induced increase in POMC production by POMC/CART-neurons is mainly carried out through the activation of the STAT3 and 3-phosphoinositide pathways, while the suppressing effect of MF on the AgRP and NPY production by AgRP/NPY-neurons is carried out through both AMPK-dependent and -independent mechanisms **[28, 39, 40, 54, 88, 89]**.

We showed that in the hypothalamus of obese agouti-mice the Thr^172^-phosphorylation of AMPK α1/2-subunit was increased, which indicates the hyperactivation of this enzyme, and MF treatment at the metabolic-improving dose (600 mg/kg/day) led to normalization of AMPK activity. It should be noted that the Ser^485/491^-phosphorylation responsible for AMPK suppression did not change significantly in all studied groups (**Fig 8**). This data indicates that MF, preventing the AMPK overstimulation, normalizes AMPK-dependent pathways in the hypothalamus, which can contribute to the improvement of the hypothalamic control of peripheral metabolism. It is important to note that more recently, Italian researchers have shown an increase in hypothalamic AMPK activity in rats with diet-induced obesity, and the increased AMPK activity was associated with oxidative stress and inflammation in the hypothalamus **[90]**.

Our results demonstrate that in the hypothalamus of MF-treated agouti-mice the STAT3 activity was decreased and did not differ significantly from control, while the Akt-kinase activity was increased as compared with both the Groups Ay/a and a/a. These results indicate that the ratio of the STAT3 and 3-phosphoinositide cascades in the hypothalamus of MF-treated agouti-mice is shifted toward the 3-phosphoinositide cascade. This is due to the restoration of the leptin and insulin content and a threefold increase in the *Lepr* expression in the hypothalamus of MF-treated mice as compared to untreated animals. The stimulating effect of MF treatment on the *Lepr* expression in the hypothalamus was previously described by other authors in diet-induced obesity **[91]**. The increase in the content of leptin and insulin in the hypothalamus of the Group Ay/a-M600 to its value in the control may be due to the MF-induced restoration of their transport through the BBB. There are numerous data on the ability of MF to restore the functions of the BBB in cerebral ischemia, sepsis and inflammation which are associated with the dysfunctions of brain microvascular endothelial cells responsible for transport of hormones and other regulators through BBB **[92–96]**.

The restoration of the activity of hypothalamic leptin system is usually associated with both an increase in the *Pomc* expression and a decrease in the *Agrp* expression **[89]**. In our case, the expression of the *Pomc* gene in the Group Ay/a-M600 was increased six times as compared with the Group a/a and three times as compared with the Group Ay/a. In turn, a slight increase in the *Agrp* expression in the Group Ay/a-M600 can be considered as a specific feature of the ASIP1-induced melanocortin obesity. There are reasons to believe that AgRP is not able to make a significant contribution to negative regulation of MC_4_R activity in the conditions of ASIP1 overproduction.

In the Group Ay/a-M600, we detected a significant decrease in the expression of the orexigenic factor NPY, which, along with POMC overexpression, can contributes to the reduced food consumption in MF-treated agouti-mice (**Figs 7 and 8**). The cause for the suppression of the *Npy* expression may be the improvement of the leptin and insulin signaling and the normalization of AMPK activity in the hypothalamus, since the expression of the *Npy* gene is negatively regulated through the leptin-, insulin- and AMPK-dependent mechanisms **[28, 40, 54, 88, 89]**.7 It should be noted that when overproduction of the MC_1/4_R-antagonist ASIP1 interferes with the inhibitory effect of AgRP, the role of NPY and other orexigenic factors acting through non-MCR-receptors in the control of eating behavior should be increased.

Using the high doses of MF, it is necessary to consider the possibility of the negative influence of MF on the liver functions, which may already be impaired in metabolic disorders, and the development of MF-induced lactic acidosis due to the altered metabolism in hepatocytes **[42–44, 97]**. We demonstrated that the 10-day treatment of agouti-mice with MF at a daily dose 600 mg/kg led to improvement of liver functions, reducing the hepatic steatosis and normalizing the expression of pro-apoptotic and pro-inflammatory factors. As noted above, the inflammation and apoptosis are the triggers for non-alcoholic fatty liver disease **[64–66]**. Previously, the other authors also showed the hepatoprotective effect of MF, which was based on a decrease in the activity of pro-apoptotic factors and on the restoration of the energy state of hepatocytes **[98, 99]**. At the same time, there is evidence that prolonged treatment with the high-dose MF induces the morphological and biochemical changes in the liver and, as a result, leads to an increase in the plasma lactate level, inducing lactic acidosis **[42]**.

We showed that in the Group Ay/a-M600, the lactate level was doubled as compared to untreated agouti-mice. However, it was below the threshold of lactate concentration (10 mM), which in rodents characterizes the development of severe forms of lactic acidosis, leading to pathological changes **[43, 44]**. We also showed the absence of the cumulative effect of MF, taken at a dose of 600 mg/kg, on the plasma levels of lactate, since there were no significant differences in the lactate concentration in agouti-mice treated with MF for one and ten days. The absence or weak expression of this effect, even in the case of the high doses of MF and its long-term administration, is due to the low lifetime of MF and its rapid removal from the blood **[100–102]**. This significantly reduces the possible risks of using high doses of MF, which was demonstrated by us in the treatment of agouti-mice and in other works where high doses of MF were used to treat mice **[103–106]**. It should be noted that the effectiveness and safety of high doses of MF largely depend on the duration of treatment, the metabolic and renal dysfunctions and the degree of liver damage **[100, 101, 107, 108]**.

## Conclusion

In agouti-mice with ASIP1-induced melanocortin-type obesity, hypothalamic signaling cascades and factors regulating food intake and metabolic processes and the compensatory mechanisms that are triggered in the response to chronic suppression of the melanocortin system was first studied. It was established that the intrahypothalamic leptin and insulin levels in obese agouti-mice was reduced due to the peripheral leptin and insulin resistance and the impaired transport of leptin and insulin through the BBB. The increase in both the *LepR* expression and the STAT3 activity was necessary to improve the response of the hypothalamic leptin system to leptin, resulting in the increased expression of the *Pomc* gene. The increased expression of genes encoding POMC and the melanocortin receptors, MC_3_R and MC_4_R, is the compensatory mechanism for prevention of ASIP1-mediated inhibition of the MC_4_R-signaling in the obese agouti-mice.

In the conditions of a malfunction of MC_4_R-signaling, the effective approaches to correct the melanocortin-type obesity are not currently developed. In this regard, a high efficacy of MF to treat obese agouti-mice is of great theoretical and practical interest. We showed that the treatment with MF (10 days, 600 mg/kg/day) resulted in the decrease of the body and fat weight, food intake and plasma levels of glucose, triglycerides and total cholesterol, all significantly increased in obese agouti-mice, and led to weakening of hyperleptinemia and hyperinsulinaemia, indicating the restoration of the leptin and insulin sensitivity. The MF treatment led to an improvement of liver functions, reducing the steatosis and the expression of the apoptotic and pro-inflammatory factors. All of the above effects of MF on the metabolic and hormonal parameters, we believe, are largely due to the restoration of the leptin, insulin and melanocortin pathways in the hypothalamus and their interaction, which demonstrates the normalization of intrahypothalamic content of leptin and insulin, the increased expression of the genes encoding LepR, POMC, MC_3_R and MC_4_R, the increased Akt-kinase activity, and, addition to this, the normalization of AMPK activity. In summary, our data suggests that MF should be considered as the effective drug for treating the melanocortin-type obesity, and the hypothalamic signaling systems and AMPK, an energy sensor within hypothalamic neurons, are one of the main targets of therapeutic action of MF.

## Supporting information

S1 Table Detailed information for antibodies used in the Western blotting experiments. (DOC)

S2 Table The sequences of the forward (For) and reverse (Rev) primers used for amplification of transcripts of target genes. (DOC)

S1 Dataset Presentation of data to the Tables 1 and 2 and to the lactate levels. (XLSX)

S2 Dataset Presentation of data to the Figs 1 and 3–7. (XLSX)

## Abbreviations

AgRP: agouti-related peptide
AMPK: AMP-activated protein kinase
ASIP1: agouti-signaling protein-1
BBB: blood-brain barrier (BBB)
GTT: glucose tolerance test
Hprt: hypoxanthine-guanine phosphoribosyl transferase
JAK2: Janus kinase-2
IRS2: insulin receptor substrate-2
LepR: leptin receptor
MC_3_R and MC_4_R: melanocortin receptors of the types 3 and 4
MF: metformin
NPY: neuropeptide Y
PI3K: phosphatidylinositol-3-kinase
POMC: pro-opiomelanocortin
STAT3: signal transducer and activator of transcription 3 (transcription factor)
TNFα: tumor necrosis factor-α
