# Supplementary material for: The evidence of metabolic-improving effect of metformin in Ay/a mice with genetically-induced melanocortin obesity and the contribution of hypothalamic mechanisms to this effect
Source: PLoS One. 2019 Mar 14;14(3):e0213779. doi: 10.1371/journal.pone.0213779 (PMC6417728; doi:10.1371/journal.pone.0213779)
Supplement: S1 Table — (DOC) [file pone.0213779.s001.doc]

**S1 Table. Detailed information for antibodies used in the Western blotting experiments.**

| **Antibody** | **Type** | **Immunogene** | **Supplier** | **Catalog #** | **Origin** | **Dilution**  **Ratio WB*** | **References** |
| --- | --- | --- | --- | --- | --- | --- | --- |
| **Primary antibodies** | | | | | | | |
| Phospho-Akt (Ser^473^) (193H12) | rabbit  monoclonal | Ser^473^ of mouse Akt | Cell Signaling Technology, Inc. | #4058 | Danvers, MA, USA | WB 1:1000 | 1, 2 |
| Phospho-Akt (Thr^308^) | rabbit  polyclonal | Thr^308^ of mouse Akt | Cell Signaling Technology, Inc. | #9275 | Danvers, MA, USA | WB 1:1000 | 3, 4 |
| Phospho-AMPKα (Thr^172^) (40H9) | rabbit  monoclonal | Thr^172^ of human AMPKα protein | Cell Signaling Technology, Inc. | #2535 | Danvers, MA, USA | WB 1:1000 | 5, 6 |
| Phospho-AMPKα1 (Ser^485^)/AMPKα2 (Ser^491^) | rabbit  policlonal | Ser^491^ of human AMPKα2 | Cell Signaling Technology, Inc. | #4185 | Danvers, MA, USA | WB 1:1000 | 7, 8 |
| Phospho-STAT3 (Tyr^705^) | rabbit  polyclonal | Tyr^705^ of mouse STAT3 | Cell Signaling Technology, Inc. | #9131 | Danvers, MA, USA | WB 1:1000 | 9, 10 |
| Akt | rabbit  polyclonal | carboxy-terminal sequence of mouse Akt | Cell Signaling Technology, Inc. | #9272 | Danvers, MA, USA | WB 1:2000 | 11, 12 |
| Stat3 (124H6) | mouse  monoclonal | human STAT3 | Cell Signaling Technology, Inc. | #9139 | Danvers, MA, USA | WB 1:1000 | 13, 14 |
| AMPK-α2 | rabbit  polyclonal | 350 and 400 of human 5’- AMPK, catalytic α2 chain  (Gene ID 5563) | Novus Biologicals | #NB100-238 | Littleton, CO, USA | WB 1:2000 | 15, 16 |
| GAPDH | mouse  monoclonal | human or rabbit GAPDH | Novus Biologicals | #NB600-502 | Littleton, CO, USA | WB 1:5000 | 17, 18 |
| **Secondary antibodies** | | | | | | | |
| anti-mouse IgG, HRP-linked | horse |  | Cell Signaling Technology, Inc. | #7076 | Danvers, MA, USA | WB:  1:1000 – 1:3000 |  |
| anti-rabbit IgG, HRP-linked | goat |  | Cell Signaling Technology, Inc. | #7074 | Danvers, MA, USA | WB:  1:1000 – 1:3000 |  |

**WB* - Western blotting**

**References:**

1. Ho MX, Poon CC, Wong KC, Qiu ZC, Wong MS. Icariin, but not genistein, exerts osteogenic and anti-apoptotic effects in osteoblastic cells by selective activation of non-genomic ERα signaling. Front Pharmacol. 2018;9:474. PMID: 29867480. PMCID: PMC5958194. doi:10.3389/fphar.2018.00474. eCollection 2018.
2. Back MJ, Ha HC, Fu Z, Choi JM, Piao Y, Won JH, et al. Activation of neutral sphingomyelinase 2 by starvation induces cell-protective autophagy via an increase in Golgi-localized ceramide. Cell Death Dis. 2018;9(6):670. PMID: 29867196. PMCID: PMC5986760. doi: 10.1038/s41419-018-0709-4.
3. Huang X, Zheng J, Li J, Che X, Tan W, Tan W, et al. Functional role of BTB and CNC Homology 1 gene in pancreatic cancer and its association with survival in patients treated with gemcitabine. Theranostics. 2018;8(12):3366-3379. PMID: 29930735. PMCID: PMC6010980. doi: 10.7150/thno.23978. eCollection2018.
4. Deodati A, Argemí J, Germani D, Puglianiello A, Alisi A, De Stefanis C, et al. The exposure to uteroplacental insufficiency is associated with activation of unfolded protein response in postnatal life. PLoS One. 2018;13(6):e0198490. PMID: 29897997. PMCID: PMC5999290. doi:10.1371/journal.pone.0198490. eCollection 2018.
5. Wang C, Qu J, Yan S, Gao Q, Hao S, Zhou D. PFK15, a PFKFB3 antagonist, inhibits autophagy and proliferation in rhabdomyosarcoma cells. Int J Mol Med. 2018;42(1):359-367. PMID: 29620138. PMCID: PMC5979828. doi: 10.3892/ijmm.2018.3599. Epub 2018 Mar 29.
6. Centini R, Tsang M, Iwata T, Park H, Delrow J, Margineantu D, et al. Loss of Fnip1 alters kidney developmental transcriptional program and synergizes with TSC1 loss to promote mTORC1 activation and renal cyst formation. PLoS One. 2018;13(6):e0197973. PMID: 29897930. PMCID: PMC5999084. doi: 10.1371/journal.pone.0197973. eCollection 2018.
7. Xia N, Weisenburger S, Koch E, Burkart M, Reifenberg G, Förstermann U, et al. Restoration of perivascular adipose tissue function in diet-induced obese mice without changing bodyweight. Br J Pharmacol. 2017;174(20):3443-3453. PMID: 28055105. PMCID: PMC5610154. doi:10.1111/bph.13703. Epub 2017 Jan 31.
8. Liu J, Wang H, Gu J, Deng T, Yuan Z, Hu B, et al. BECN1-dependent CASP2 incomplete autophagy induction by binding to rabies virus phosphoprotein. Autophagy. 2017;13(4):739-753. PMID: 28129024. PMCID: PMC5388250. doi: 10.1080/15548627.2017.1280220. Epub 2017 Jan 27.
9. Mayoral-Varo V, Calcabrini A, Sánchez-Bailón MP, Martín-Pérez J. miR205 inhibits stem cell renewal in SUM159PT breast cancer cells. PLoS One. 2017;12: e0188637. PMID: 29182685. PMCID: PMC5705145. doi: 10.1371/journal.pone.0188637.
10. Hadjidaniel MD, Muthugounder S, Hung LT, Sheard MA, Shirinbak S, Chan RY, et al. Tumor-associated macrophages promote neuroblastoma via STAT3 phosphorylation and up-regulation of c-MYC. Oncotarget. 2017;8(53):91516-91529. PMID: 29207662. PMCID: PMC5710942. doi: 10.18632/oncotarget.21066. eCollection 2017 Oct 31.
11. Panse M, Kluth O, Lorza-Gil E, Kaiser G, Mühlbauer E, Schürmann A, et al. Palmitate and insulin counteract glucose-induced thioredoxin interacting protein (TXNIP) expression in insulin secreting cells via distinct mechanisms. PLoS One. 2018;13(5):e0198016. PMID: 29813102. PMCID: PMC5973613. doi:10.1371/journal.pone.0198016. eCollection 2018.
12. Ponnusamy A, Sinha S, Hyde GD, Borland SJ, Taylor RF, Pond E, et al. FTI-277 inhibits smooth muscle cell calcification by up-regulating PI3K/Akt signaling and inhibiting apoptosis. PLoS One. 2018;13(4):e0196232. PMID: 29689070. PMCID: PMC5916518. doi: 10.1371/journal.pone.0196232. eCollection 2018.
13. Kulling PM, Olson KC, Hamele CE, Toro MF, Tan SF, Feith DJ, et al. Dysregulation of the IFN-γ-STAT1 signaling pathway in a cell line model of large granular lymphocyte leukemia. PLoS One. 2018;13(2):e0193429. PMID: 29474442. PMCID: PMC5825082. doi: 10.1371/journal.pone.0193429. eCollection 2018.
14. Mainardi M, Spinelli M, Scala F, Mattera A, Fusco S, D'Ascenzo M, et al. Loss of leptin-induced modulation of hippocampal synaptic trasmission and signal transduction in high-fat diet-fed mice. Front Cell Neurosci. 2017;11:225. PMID: 28804449. PMCID: PMC5532388. doi: 10.3389/fncel.2017.00225. eCollection 2017.
15. Jaitovich A, Angulo M, Lecuona E, Dada LA, Welch LC, Cheng Y, et al. High CO2 levels cause skeletal muscle atrophy via AMP-activated kinase (AMPK), FoxO3a protein, and muscle-specific Ring finger protein 1 (MuRF1). J Biol Chem. 2015;290(14):9183-94. PMID:25691571. PMCID: PMC4423704. doi: 10.1074/jbc.M114.625715. Epub 2015 Feb 17.
16. Gusarova GA, Dada LA, Kelly AM, Brodie C, Witters LA, Chandel NS, et al. Alpha1-AMP-activated protein kinase regulates hypoxia-induced Na,K-ATPase endocytosis via direct phosphorylation of protein kinase C zeta. Mol Cell Biol. 2009;29(13):3455-64. PMID: 19380482. PMCID: PMC2698765. doi: 10.1128/MCB.00054-09. Epub 2009 Apr 20.
17. Tacconi EM, Lai X, Folio C, Porru M, Zonderland G, Badie S, et al. BRCA1 and BRCA2 tumor suppressors protect against endogenous acetaldehyde toxicity. EMBO Mol Med. 2017;9(10):1398-1414. PMID: 28729482. PMCID: PMC5623864. doi: 10.15252/emmm.201607446.
18. Zhang P, Tsuchiya K, Kinoshita T, Kushiyama H, Suidasari S, Hatakeyama M, et al. Vitamin B6 Prevents IL-1β protein production by inhibiting NLRP3 inflammasome activation. J Biol Chem. 2016;291(47):24517-24527. Epub 2016 Oct 12. PMID: 27733681. PMCID: PMC5114405.
