# Supplementary material for: The evidence of metabolic-improving effect of metformin in Ay/a mice with genetically-induced melanocortin obesity and the contribution of hypothalamic mechanisms to this effect
Source: PLoS One. 2019 Mar 14;14(3):e0213779. doi: 10.1371/journal.pone.0213779 (PMC6417728; doi:10.1371/journal.pone.0213779)
Supplement: S2 Table — (DOC) [file pone.0213779.s002.doc]

**S2 Table. The sequences of the forward (For) and reverse (Rev) primers used for amplification of transcripts of target genes.**

| No | Gene | Localization | Primer | NCBI Reference Sequence |
| --- | --- | --- | --- | --- |
| 1 | *Pomc* | For 5’–3’ | CAGTGCCAGGACCTCACC | NM_008895.4 |
|  |  | Rev 5’–3’ | CAGCGAGAGGTCGAGTTTG |  |
| 2 | *Agrp* | For 5’–3’ | ACAACTGCAGACCGAGCAGAA | NM_001271806.1 |
|  |  | Rev 5’–3’ | CGACGCGGAGAACGAGACT |  |
| 3 | *Npy* | For 5’–3’ | CGGGAGAACAAGTTTCATTTCC | NM_023456.3 |
|  |  | Rev 5’–3’ | ACAGAAAACGCCCCCAGAAC |  |
| 4 | *Lepr* | For 5’–3’ | GCATGCAGAATCAGTGATATTTGG | NM_146146.2 |
|  |  | Rev 5’–3’ | CAAGCTGTATCGACACTGATTTCTTC |  |
| 5 | *Mc3r* | For 5’–3’ | CAAGGAGATTCTCTGCGGCT | NM_008561.3 |
|  |  | Rev 5’–3’ | TCCCGTCTGAGCGTTGTTTT |  |
| 6 | *Mc4r* | For 5’–3’ | GGGTCGGAAACCATCGTCAT | NM_016977.4 |
|  |  | Rev 5’–3’ | TGCAAATGGATGCGAGCAAG |  |
| 7 | *Bax* | For 5’–3’ | TGGAGCTGCAGAGGATGATTG | NM_007527.3 |
|  |  | Rev 5’–3’ | GAAGTTGCCGTCAGAAAACATG |  |
| 8 | *Bcl2* | For 5’–3’ | GTGGATGACTGAGTACCTGAAC | NM_009741.5 |
|  |  | Rev 5’–3’ | GAGACAGCCAGGAGAAATCAA |  |
| 9 | *IL1beta* | For 5’–3’ | TCCAGGATGAGGACATGAGCAC | NM_008361.4 |
|  |  | Rev 5’–3’ | GAACGTCACACACCAGCAGGTTA |  |
| 10 | *TNFalpha* | For 5’–3’ | TATGGCCCAGACCCTCACA | NM_013693.3 |
|  |  | Rev 5’–3’ | GGAGTAGACAAGGTACAACCCATC |  |
| 11 | *Hprt* | For 5’–3’ | AGCCGACCGGTTCTGTCAT | NM_013556.2 |
|  |  | Rev 5’–3’ | GGTCATAACCTGGTTCATCATCAC |  |
| 12 | *18S rRNA* | For 5’–3’ | GGGAGCCTGAGAAACGGC | NR_003278.3 |
|  |  | Rev 5’–3’ | GGGTCGGGAGTGGGTAATTT |  |
